# Medical Items

**Published:** 1894-05

**Authors:** 


### *MEDICAL ITEMS.*

### FEE BILL ADOPTED BY THE SOUTH GEORGIA MEDICAL ASSOCIATION, SEPTEMBER, 1893.

### SURGERY.

| | | | |
|---|---|---|---|
| Dressing contused, lacerated or incised wounds | $ 1 00 | to $ | 5 00 |
| Catheterization | 1 00 | " | 5 00 |
| Opening abscess or boil | | | 1 00 |
| Extracting teeth | | | 1 00 |
| Amputation fingers and toes | | | 5 00 |
| Amputation of arm, forearm or leg | 25 00 | " | 50 00 |
| Amputation of thigh | 50 00 | " | 150 00 |
| Tapping hydrocele | | | 5 00 |
| Paracentesis abdominis | 5 00 | " | 20 00 |
| Reducing dislocation | 5 00 | " | 25 00 |
| Reducing fracture | 5 00 | " | 50 00 |
| Ligation of arteries | 5 00 | " | 25 00 |
| Anesthesia extra | | | 5 00 |

### GYNECOLOGY.

| | | | |
|---|---|---|---|
| Examination (rectal or vaginal) digital or speculum, including applications to os uteri | | | 2 50 |
| Tamponing vagina | | | 5 00 |
| Dilating os uteri | 25 00 | " | 50 00 |

### MIDWIFERY.

| | | | |
|---|---|---|---|
| Obstetrics uncomplicated in town (detention at discretion of physician) | | | 10 00 |
| For extra babies, each (mileage to be added in obstetrics as in all other practice) | | | 5 00 |
| Forceps delivery | 25 00 | " | 50 00 |
| Version | | | 25 00 |
| Delivering placenta where child is found born, mileage added | | | 5 00 |

### GENERAL OFFICE CALL.

| | | | |
|---|---|---|---|
| Consultation of office patient | 1 00 | " | 5 00 |
| Prescription at office, medicine added | | | 1 00 |
| Consultation, physician, mileage added | | | 10 00 |
| Visit and prescription in town, daytime, medicine added | | | 2 00 |
| Night-time, medicine added | 2 00 | " | 5 00 |
| Mileage in the day | 50c | " | 1 00 |
| Mileage in the night | 1 00 | " | 2 00 |

### AMERICAN SURGICAL ASSOCIATION—SESSION OF 1894.

### SPECIAL SUBJECTS FOR DISCUSSION.

I. "Surgical Treatment of Empyema," by John Ashurst, Jr., M. D.

The discussion of the paper will be opened by Drs. N. P. Dandridge, C. B. Dancrede, T. F. Pruitt and DeF. Willard.

II. "Methods of Teaching Surgery," by J. S. Billings, M. D.

The discussion of the paper will be opened by Drs. J. C. Warren, N. Senn, W. W. Keen, E. M. Moore, W. T. Briggs and Hunter McGuire. It is desired that the discussion of this paper should be participated in by the Fellows generally.

III. "The Surgery of the Kidney," by L. M. Tiffany, M. D.

The discussion of the paper will be opened by Drs. M. H. Richardson, H. H. Mudd, C. H. Mastin and Ford Thompson.

IV. "Methods of Controlling Hemorrhage in Amputation at the Shoulder," as illustrated by three cases of amputation at the shoulder-joint and of the entire upper extremity, by W. W. Keen, M. D.

The discussion of the paper will be opened by Drs. Roswell Park, C. B. Porter and J. William White.

V. Paper by Hunter McGuire, M. D.

VI. Paper by Joseph Ransohoff, M. D.

Fellows who desire to present volunteer papers are requested to send the titles of the papers to the address of the Business Committee, 1429 Walnut Street, Philadelphia, not later than April 18th, 1894.

The sessions of the Association will be held in the Lecture-room of the Medical Department of the Columbia College, 1325 H Street, N. W., Washington, D. C., on May 29th, 30th, 31st, and June 1st, from 10 A. M. to 1 P. M.

The sessions of the Congress will be held in Metzerott's Hall, corner of Twelfth and F Streets, N. W., in the afternoon from two to five o'clock.

The office of registration of the Congress will be at the Arlington Hotel. Registration of individual members of the constituent associations of the Congress is necessary to make them members of the Congress.

Dinner to the guests of the Congress at the Arlington Hotel, Wednesday, May 30th, 7 P. M.

Reception by the President of the Congress, Thursday evening, May 31st.

J. R. WEIST, M.D., Secretary.

